# Skimmianine Showed Neuroprotection against Cerebral Ischemia/Reperfusion Injury

**DOI:** 10.3390/cimb46070437

**Published:** 2024-07-12

**Authors:** Hayat Ayaz, Fırat Aşır, Tuğcan Korak

**Affiliations:** 1 Department of Histology and Embryology, Medical Faculty, Dicle University, 21280 Diyarbakır, Turkey; 2 Department of Medical Biology, Medical Faculty, Kocaeli University, 41001 Kocaeli, Turkey; tugcankorak@gmail.com

**Keywords:** natural products, bioactive compounds, phytochemistry, molecular science

## Abstract

The aim of this study was to investigate the antioxidant and anti-inflammatory effects of skimmianine on cerebral ischemia–reperfusion (IR) injury. Twenty-four female Wistar albino rats were randomly divided into three groups: Sham, Ischemia–Reperfusion (IR), and IR + Skimmianine (40 mg/kg Skimmianine). Cerebral ischemia was induced using a monofilament nylon suture to occlude the middle cerebral artery for 60 min. Following 23 h of reperfusion, the animals were sacrificed 14 days later. The effects of skimmianine on brain tissue post-IR injury were examined through biochemical and immunochemical analyses. In silico analysis using the Enrichr platform explored skimmianine’s potential biological processes involving IBA-1, IL-6, and NF-κB proteins. In the IR group, MDA levels increased, while SOD and CAT antioxidant enzyme activities decreased. In the IR + Skimmianine group, skimmianine treatment resulted in decreased MDA levels and increased SOD and CAT activities. Significant increases in IBA-1 expression were observed in the IR group, which skimmianine treatment significantly reduced, modulating microglial activation. High levels of IL-6 expression were noted in pyramidal neurons, vascular structures, and neuroglial cells in the IR group; skimmianine treatment reduced IL-6 expression, demonstrating anti-inflammatory effects. Increased NF-κB expression was observed in neurons and blood vessels in the gray and white matter in the IR group; skimmianine treatment reduced NF-κB expression. Gene Ontology results suggest skimmianine impacts immune and inflammatory responses via IBA-1 and IL-6, with potential effects on estrogen mechanisms mediated by NF-κB. Skimmianine may be a potential therapeutic strategy due to its antioxidant and anti-inflammatory effects on cerebral IR injury.

## 1. Introduction

Cerebral stroke is a significant cause of morbidity and mortality that significantly affects healthcare systems in developing countries [1,2,3,4]. It affects approximately 13.7 million people annually and leads to around 5.5 million deaths [5]. Cerebral stroke refers to the interruption of local blood flow in the brain, which typically occurs as a result of conditions such as cerebral vascular rupture or arterial embolism [6]. This condition can trigger various neurological complications such as brain edema, epilepsy, recurrent strokes, and delirium [7]. Common treatment of cerebral stroke is ensuring reperfusion [6]. However, initiating reperfusion can potentially lead to cerebral ischemia/reperfusion (IR) injury [3]. Stroke can be induced by different causes such as blood clot due to thrombosis, embolism [8], or hemorrhage due to aneurysm, arteriovenous malformation, hypertension and trauma [9]. Risk factors for stroke include high blood pressure, diabetes, high cholesterol, smoking and alcohol consumption, obesity, and increased age [10]. The pathophysiology of cerebral IR injury is quite complex, involving oxidative stress, inflammatory response, and cell death [11]. During oxidative stress, reactive oxygen species (ROS) are released from mitochondria, enzymatic reactions, and inflammatory cells, leading to cellular and tissue damage in cells [12,13]. Formation of ROS increases lipid peroxidation in cell membranes, disrupting membrane structure and integrity. Moreover, ROS production causes protein misfolding and degradation, as well as genomic instability. As a consequence, chronic oxidative stress causes cell death, chronic inflammation, and neurodegenerative disease in the long-term period [12,14].

Cerebral IR injury is a significant factor that contributes to adverse outcomes following reperfusion therapy in patients with ischemic stroke [15]. Although reperfusion of the ischemic brain seems to be curative for the infarct area, it paradoxically causes damage [16]. The reason behind this phenomenon is production of ROS (due to sudden influx of oxygen), induction of inflammation (activation of inflammatory mediators), disruption of blood brain barrier, excitotoxicity, calcium overload, mitochondrial dysfunction, vascular injury (endothelial cell damage), microvascular obstruction, and cellular death pathways (apoptosis and necrosis) [15,16,17]. Taking all these into consideration, reperfusion therapy is essential for limiting the extent of brain damage in ischemic stroke, but it also introduces a complex cascade of events that can cause additional injury to the brain.

Neuroinflammation is known as an inflammatory response in the central nervous system and is considered one of the key mechanisms of cerebral IR injury [18,19]. There is still no effective neuroprotective treatment available that effectively preserves the central nervous system during the process of cerebral IR injury [20]. The active compounds found in medicinal plants contribute to the treatment, prevention, and drug development processes of diseases [21].

The effects of components found in plant extracts such as formononetin [22], salvianolic acid C [23], curcumin [24], syringin [25], and noscapine [26] have been investigated in the treatment of cerebral ischemia–reperfusion (IR). However, there have been no reports of studies investigating the anti-neuroinflammatory and antioxidative effects of skimmianine on cerebral IR injury in the literature. Skimmianine is a furoquinoline alkaloid commonly found in plants of the Rutaceae family, and chemically referred to as 4,7,8-trimethoxyfuro [2,3-b] quinoline [27]. It has various pharmacological activities such as antioxidant, anti-inflammatory, antiplatelet aggregation, antiproliferative, analgesic, and antiviral properties. This component demonstrates its anti-inflammatory potential by regulating the activation of receptor proteins involved in inflammatory signaling pathways [27,28,29]. It provides its antioxidant activity by reducing lipid peroxidation and increasing the activity of natural antioxidant enzymes. Skimmianine exhibits anti-inflammatory effects through activation of the phosphatidylinositol-3-kinase (PI3K)–protein kinase B (AKT) pathway in liver ischemia–reperfusion (IR) injury, and demonstrates antioxidant activity by reducing lipid peroxidation and increasing the activity of antioxidant enzymes [28]. Results from a study investigating the neuroprotective effect of skimmianine in lipopolysaccharide-stimulated BV-2 microglial cells revealed that skimmianine targets the NF-κB activation pathway to prevent neuroinflammation [30]. There is limited information about dosage of skimmianine treatment across different species. Huo et al. [28] investigated dosage-dependent use of skimmianine on male mice against liver ischemia–reperfusion injury. The authors suggested that protective effects of skimmianine were most obvious at a dose of 40 mg/kg (vs. 10, 20, and 80 mg/kg) administration by lowering level of hepatic damage enzymes, necrotic area, alleviating inflammation response, oxidative stress, and apoptosis.

Microglia, one of the key mediators of the immune response, become markedly activated in the central nervous system following cerebral injury; moreover, microglial activation is considered a pivotal indicator of neuroinflammation [19]. Ionized calcium-binding adapter molecule 1 (IBA-1) is a cytoplasmic protein found in microglia in cerebral tissue [31]. He et al. [32] stated that the increased expression of the IBA-1 protein in brain tissue during experimental cerebral ischemia/reperfusion (IR) injury is associated with an inflammatory response and reflects microglial activation.

NF-κB is a transcription factor that serves as a key regulator of cellular signaling pathways. Additionally, it has a tight association with immune response and inflammation. Activation of NF-κB can lead to the production of proinflammatory cytokines; this plays a key role in regulating immune response and inflammation processes [33]. In an experimental study, it was determined that the expression of NF-κB protein increased in brain tissue during cerebral ischemia/reperfusion (IR) injury. The results of the study showed that the activation of the NF-κB signaling pathway led to microglial hyperactivation, resulting in increased production of proinflammatory cytokines and exacerbation of tissue damage [34].

Interleukin 6 (IL-6) is a proinflammatory cytokine produced by monocytes and macrophages that plays a role in central immune defense [35]. It is known to be effective in the neuroinflammation process that occurs during cerebral ischemia–reperfusion injury [36]. Shi et al. identified that during experimental cerebral ischemia/reperfusion (IR) injury, the NF-κB signaling pathway is activated in brain tissue associated with an inflammatory response. The study revealed that this activation leads to an increase in the expression of proinflammatory cytokines such as IL-6, contributing to brain damage [37].

There seems to be a gap regarding the role of skimmianine on neural tissue. The aim of this study is to investigate the antioxidant and anti-inflammatory properties of skimmianine in cerebral IR injury via histochemical, immunohistochemical, and in silico analysis.

## 2. Results

### 2.1. Skimmianine Favored the Antioxidant Mechanism

**Figure 1:**
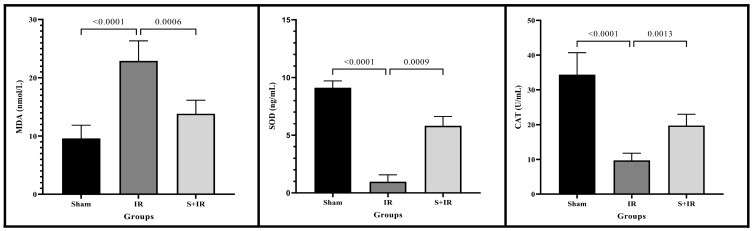
Effects of skimmianine on oxidative parameters of in group.

Blood samples were analyzed for MDA, SOD, and CAT activities. MDA is the end product of lipid peroxidation and is used as a marker for oxidative stress levels. Following cerebral ischemia–reperfusion (IR) injury, MDA content significantly increased in the IR group compared to the sham group. SOD and CAT levels were used to demonstrate cellular antioxidant activity. After cerebral ischemia–reperfusion induction, both SOD and CAT levels markedly decreased in the IR group compared to the sham group. Due to its antioxidant effects, skimmianine led to a reduction in MDA levels and an increase in SOD and CAT activities (Figure 1).

### 2.2. Skimmianine Modulates Microglial Activity and Protects Cerebral Tissue

**Figure 2:**
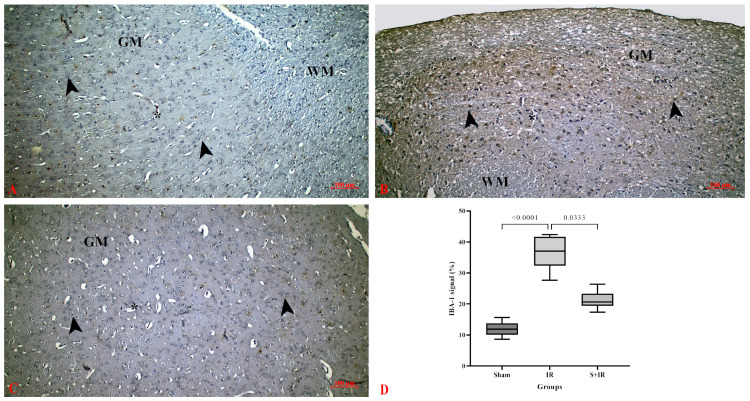
Immunoexpression of IBA-1 in cerebral tissues. (**A**) Sham group, (**B**) IR group, (**C**) S + IR group, (**D**) histological scoring of immunoexpression; arrowhead: neuron, GM: gray matter, WM: white matter, asterisk: capillary.

IBA-1 immune activities in cerebral sections are shown in Figure 2. IBA-1 immune staining was performed to show microglial activity in immune defense after cerebral IR injury. In the control group, IBA-1 expression in gray matter and white matter was generally negative; however, IBA-1 expression was positively observed in some areas of the cerebral cortex and in the blood vessels (Figure 2A). A significant increase in IBA-1 expression was observed after cerebral IR injury, with microglia in the cerebral cortex, blood vessels, and white matter expressing IBA-1 (Figure 2B). After skimmianine treatment, IBA-1 expression significantly decreased (Figure 2C). Semi-quantitative analysis showed that skimmianine significantly downregulated the expression of IBA-1 in cerebral tissues (Figure 2D).

### 2.3. Skimmianine Prevents Neuroinflammation by Downregulating IL-6 Expression

**Figure 3:**
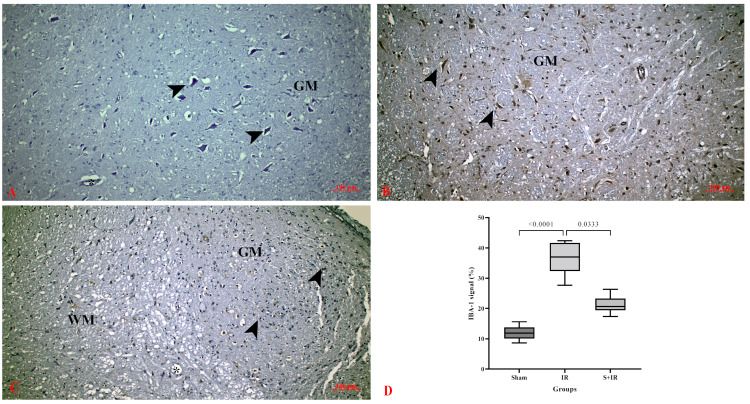
Immunoexpression of IL-6 in cerebral tissues. (**A**) Sham group, (**B**) IR group, (**C**) S + IR group, (**D**) histological scoring of immunoexpression; arrowhead: neuron, GM: gray matter, WM: white matter, asterisk: capillary.

IL-6 immune staining was performed to show the inflammatory response after cerebral IR injury. In the control group, neurons and neuroglia in the cerebral cortex and white matter did not express IL-6 (Figure 3A). After cerebral IR injury, a high level of IL-6 expression was observed in pyramidal neurons, vascular structures, and neuroglial cells in gray matter. The expression was increased in the IR group compared to the control group (Figure 3B). Skimmianine downregulated the expression of IL-6 in cells in both white and gray matter, with IL-6 expression mainly negative in the IR + Skimmianine group (Figure 3C). Semi-quantitative analysis showed that skimmianine significantly downregulated IL-6 expression in cerebral tissues (Figure 3D).

### 2.4. Skimmianine Targets Key Mediators of Neuroinflammation by Downregulating NF-κB Expression

**Figure 4:**
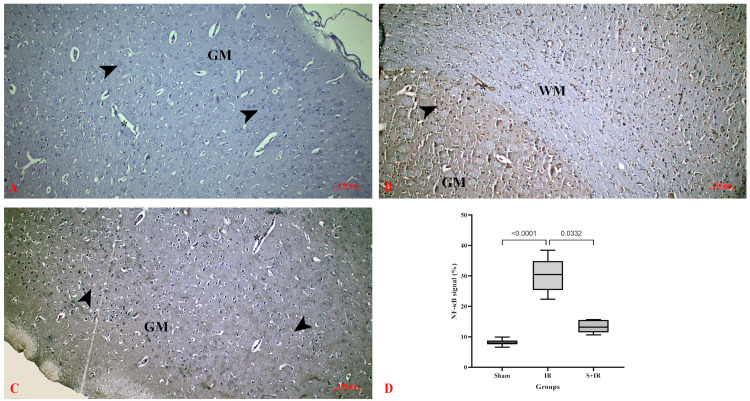
Immunoexpression of NF-κB in cerebral tissues. (**A**) Sham group, (**B**) IR group, (**C**) S + IR group, (**D**) histological scoring of immunoexpression; arrowhead: neuron, GM: gray matter, WM: white matter, asterisk: capillary.

NF-κB is a complex protein activated in many cellular conditions such as inflammation and oxidative stress. In the control group, NF-κB expression was negative in cerebral neurons, neuroglia, and vessels in gray matter (Figure 4A). Cerebral IR injury caused upregulation of NF-κB expression in neurons and vessels in gray matter, and mostly in vessels and neuroglia in white matter, with higher expression in the IR group compared to the control group (Figure 4B). Administration of Skimmianine prevented the activation of NF-κB in neurons and glial cells, lowering NF-κB expression in the IR + Skimmianine group compared to the IR group (Figure 4C). Semi-quantitative measurements showed that skimmianine significantly decreased NF-κB immune reactivity in cerebral tissues (Figure 4D).

### 2.5. Annotated Gene Ontology, Skimmianine, IBA-1, IL-6, and NF-κB

**Figure 5:**
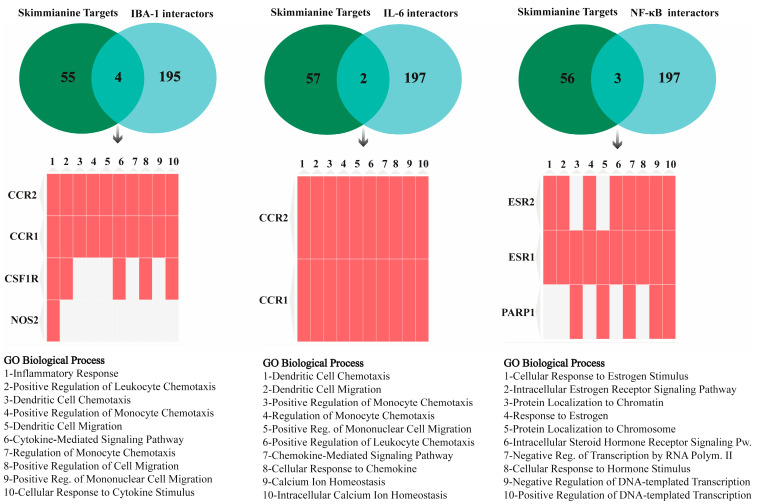
GO Biological Process analysis for shared targets of skimmianine, IBA-1, IL-6, and NF-κB. The Venn diagram illustrates the number of interactors. The *x*-axis of the three matrices (1–10) represents enriched terms, while the *y*-axis represents input genes. The cells in the matrix indicate the association status with a term. They are ranked according to the *p*-values of the top ten enriched GO Biological Process terms, and significant annotations (*p* < 0.05) are represented.

Upon scanning databases to identify skimmianine’s target proteins, fifty-nine, nineteen, and five target proteins were identified in SwissTargetPrediction, ChEMBL, and PubChem databases, respectively. However, no target proteins for Skimmianine were identified in STITCH and DrugBank databases. When examining the common interactors of Skimmianine with IBA-1, IL-6, and NF-κB, only the SwissTargetPrediction database yielded shared proteins. The common targets of skimmianine with IBA-1 include CCR1, CCR2, CSF1R, and NOS2. The common targets with IL-6 include CCR1 and CCR2, and the common targets with NF-κB include ESR1, ESR2, and PARP1. The top 10 annotations from the GO Biological Process analysis of the shared targets are listed in Figure 5.

## 3. Discussion

This study was conducted to evaluate the protective effects of skimmianine on cerebral I/R injury. The results provide important findings regarding the antioxidant and anti-inflammatory potential of skimmianine. Cerebral I/R injury is a key factor contributing to the adverse outcomes observed following reperfusion therapy in patients with ischemic stroke. However, currently, effective neuroprotective treatments targeting cerebral I/R injury are limited [38]. This study showed that skimmianine is effective in reducing cerebral I/R injury.

Oxidative stress, resulting from the increase of reactive oxygen species, is considered a fundamental mechanism in the development of cerebral I/R injury [39]. This may contribute to mitochondrial dysfunction, exacerbating I/R injury [28]. Therefore, oxidative stress is seen as an important critical target point in treatment [39]. One of the mechanisms underlying the protective effects of skimmianine is its antioxidant activity. In an experimental study, skimmianine treatment effectively counteracted oxidative stress by enhancing the activities of antioxidant enzymes such as SOD, CAT, and glutathione peroxidase (GPx) through its antioxidant properties [40]. Huo et al. [28] determined that skimmianine treatment reduced oxidative stress in hepatic I/R injury by increasing SOD and GPx antioxidant enzyme activities and decreasing MDA levels. In our study, skimmianine treatment for cerebral I/R injury increased the low levels of CAT and SOD enzyme activities while decreasing the high levels of MDA. Our findings indicate that skimmianine alleviates oxidative damage by neutralizing free radicals, thus reducing cerebral I/R injury.

Cerebral I/R injury causes activation of various cellular signaling molecules and immune cells. This leads to the onset of the inflammatory response and the emergence of neuroinflammation. It can be thought that neuroinflammation in the first stage of cerebral I/R injury is a defense mechanism to protect brain tissue. However, excessive and prolonged neuroinflammation can worsen the effects of ischemic stroke by increasing cell and tissue damage [41].

Microglial cells are innate immune cells that reside in a quiescent state in the central nervous system [42]. They are activated in neuropathological conditions, including ischemic stroke. Activated microglia undergo morphological and phenotypic changes [42,43]. Microglial activation exacerbates cerebral tissue damage in ischemic stroke by leading to the release of pro-inflammatory cytokines such as interleukin-1 beta (IL-1β), IL-6, and tumor necrosis factor-alpha (TNF-α) [42,44,45]. IBA1 expression, a microglial marker in cerebral tissues, can indicate microglial activation in cerebral I/R injury [46]. Our study demonstrated that treatment with skimmianine reduced microglial activation by decreasing the increased expression of IBA-1 protein induced by cerebral I/R injury.

NF-κB signaling plays a critical role in regulating inflammatory responses in the brain following ischemic stroke [41]. Nuclear translocation of NF-κB occurs in ischemic stroke [43]. Activation of NF-κB signaling leads to the release of inflammatory mediators such as IL-18, IL-6, and TNF-α [42]. Furthermore, activation of NF-κB signaling leads to a phenotypic change in microglia, resulting in an increased inflammatory response. This contributes to the exacerbation of tissue damage [42,43]. Chen et al. found that NF-κB, IBA-1, and IL-6 protein expressions increased in brain tissue in cerebral I/R. The authors stated that the transcription activity of NF-κB in cerebral I/R injury leads to excessive microglial activation and that microglial activation increases cerebral tissue damage by significantly increasing the production of pro-inflammatory cytokines TNF-α, IL-6, and IL-1β [34]. Suppression of the NF-κB signaling pathway and microglial activation in cerebral I/R injury can be considered a possible therapeutic strategy to ameliorate cerebral tissue damage. There are studies showing skimmianine’s ability to regulate the activities of immunoregulatory cytokines and enzymes in the inflammatory response [28,40].

In a study evaluating the anti-inflammatory activity of skimmianine in carrageenan-induced rat paw edema, it was found that mRNA levels of inflammatory mediators such as TNF-α and IL-6 were reduced [40]. In an experimental study, it was determined that skimmianine showed an anti-inflammatory effect in liver I/R injury by inhibiting the increased levels of pro-inflammatory cytokines such as TNF-α, IL-6, and IL-1β in liver tissue [28]. In an LPS-induced neuroinflammation study in the BV-2 microglia cell line, the effects of skimmianine on the production of pro-inflammatory mediators were investigated. Skimmianine treatment was proven to reduce the levels of pro-inflammatory cytokines such as TNF-α and IL-6 in cell supernatants and inhibit neuroinflammation by targeting the NF-κB activation pathway [30]. Our findings indicate that skimmianine treatment reduces IL-6 and NF-κB protein expressions in brain tissue following cerebral I/R injury. These results strongly suggest that skimmianine may have anti-inflammatory effects in cerebral I/R injury, highlighting its potential therapeutic value in mitigating neuroinflammation.

In our experimental findings, we demonstrated the antioxidant and anti-inflammatory effects of skimmianine in rats subjected to cerebral ischemia/reperfusion injury, alongside the observed suppression of IBA-1, IL-6, and NF-κB protein levels. Subsequently, we conducted GO biological process analysis on the shared targets of skimmianine and these three proteins to elucidate which pathways skimmianine may affect through IBA-1, IL-6, and NF-κB, potentially contributing to its anti-inflammatory and antioxidant effects. For this purpose, we investigated the skimmianine targets across multiple databases. The absence of target proteins for skimmianine in STITCH and DrugBank databases suggests either a lack of information or different methodologies used in these databases for predicting drug-target interactions. Therefore, the importance of considering multiple databases and methodologies when identifying drug target proteins is highlighted. Our in silico analysis revealed several enriched GO biological process terms for IBA-1 and IL-6, including inflammatory response, dendritic cell chemotaxis/migration, leukocyte chemotaxis, cytokine-mediated signaling pathway, and cellular response to cytokine stimulus, among others. These results suggest that skimmianine may exert its effects on reducing cerebral ischemia/reperfusion injury through modulation of inflammatory and immune responses via IBA-1 and IL-6. Experimental studies have also shown that IBA-1 plays a key role in inflammatory processes and the development of the immune response by engaging in proinflammatory activity [47], triggering chemokine production [48], macrophage and microglial activation, and dendritic cell differentiation and function [49,50]. In addition, it has been found that IBA-1 also influences the production of IL-6, which is produced by dendritic cells and affects important pathways such as JAK/STAT and TGF-β, leading to both pro-inflammatory and anti-inflammatory activity according to the immune response [50,51]. On the other hand, it was found that the NF-κB-associated targets of skimmianine notably involve cellular response to estrogen stimulus and estrogen receptor-associated signaling pathways. Estrogens possess antioxidant properties by binding to intracellular estrogen receptors (estrogen receptor α and estrogen receptor β) and enhancing the expression of antioxidant enzymes through intracellular signaling pathways [52]. The hormone-receptor complex translocates the nucleus of a cell and modulates transcription of target genes, causing a change in protein synthesis [53,54]. In addition to gene regulation, estrogen can directly alter signaling pathways, activating many kinases and other signaling molecules, leading to rapid cellular response [54,55,56]. Therefore, given that estrogen has many impacts on the reproductive system, its action is also important for the non-reproductive system. The G protein-coupled estrogen receptor 1 (GPER) is an estrogen receptor responsible for regulating various signaling pathways in the brain. Endogenously activated by estrogen, GPER influences cell migration, proliferation, apoptosis, cellular balance, neuronal health, and neurogenesis across different brain regions. GPER modulates cellular responses through intricate signaling cascades such as the PI3K/Akt/Gsk-3β and ERK1/2 pathways, thereby exerting regulatory effects on oxidative stress and neuroinflammation [57]. Therefore, estrogen-associated regulatory mechanisms are important for both antioxidant and inflammation mechanisms in the brain. Collectively, our study suggests that skimmianine may alleviate cerebral ischemia/reperfusion injury by modulating immune and inflammatory responses, as well as antioxidant mechanisms mediated by IBA-1, IL-6, and NF-κB.

In our study, we determined that the increased NF-κB, IBA-1, and IL-6 protein expressions in cerebral tissue after cerebral I/R injury were reduced by skimmianine treatment. Our findings indicate that the anti-inflammatory effect of skimmianine in cerebral I/R injury is achieved through the suppression of microglia activation and the reduction of pro-inflammatory cytokine expression as a result of the inhibition of the NF-κB signaling pathway.

## 4. Materials and Methods

### 4.1. Experimental Design

This study was approved by the Animal Experiments Local Ethics Committee, Dicle University (approval number: 2023/31). In the experiment, 24 female Wistar albino rats, each weighing between 240–260 g and aged 10–12 weeks, were used. The rats were housed in standard cages under a 12 h light/dark cycle at a room temperature of 23 ± 2 °C. Throughout the study, they were provided with a standard rat pellet diet and had unrestricted access to water. Skimmianine (catalog no: HY-N2081, MedChemExpress, Shanghai, China) and sodium carboxymethyl cellulose (catalog no: CAS 9004-32-4, Santa Cruz Biotechnology Inc., Dallas, TX, USA) were commercially purchased.

### 4.2. Induction of Cerebral IR Injury and Experimental Groups

All surgical procedures were conducted under general anesthesia. Rats were placed in the supine position, and an approximately 3 cm midline incision extending from the upper edge of the sternum to the hyoid bone was made. The incision area was expanded, and the trachea was exposed through blunt dissection. Paratracheal muscles were dissected, reaching the common carotid artery (CCA). Entry into the lumen of the internal carotid artery was achieved using a 4-0 monofilament nylon suture, advanced to occlude the middle cerebral artery (MCA). Ischemia was induced for 60 min; then, the suture was removed, and the surgical procedure was concluded. Subsequently, a 23 h reperfusion was established [30].

Sham (*n* = 8): Rats were intraperitoneally administered 1 cc 5% sodium carboxymethyl cellulose solution for 14 days. The left CCA was isolated and followed by fascia and skin closure. Animals were then taken into their cages.

Ischemia–Reperfusion (IR) (*n* = 8): Rats were intraperitoneally administered a 1 cc solution containing 5% sodium carboxymethyl cellulose for 14 days. Subsequently, cerebral ischemia was induced. The animals were then placed in their cages for the reperfusion process.

Ischemia–Reperfusion (IR) + Skimmianine (*n* = 8): Rats were intraperitoneally administered 40 mg/kg skimmianine dissolved in a 1 cc solution containing 5% sodium carboxymethyl cellulose for 14 days [21]. After inducing cerebral ischemia, the animals were allowed to rest in their cages for the reperfusion process.

After the experimental protocol was finished, rats were euthanized under general anesthesia. Blood samples were collected, and the brain was excised from each animal.

### 4.3. Determination of MDA Content, SOD and CAT Activities

Malondialdehyde (MDA, #MAK085, Merck KGaA, Darmstadt, Germany), Superoxide Dismutase (SOD) (Cat. No: MBS2707324, MyBioSource, San Diego, CA, USA), and Catalase (CAT) (Cat. No: MBS726781, MyBioSource, San Diego, CA, USA) for colorimetric analysis were commercially purchased. Blood samples from each rat were centrifuged at 2000 rpm for 10 min, and the supernatant was collected. Serum plasma from the blood samples was separately analyzed for MDA (nmol/mL) content, SOD (ng/mL), and CAT (ng/mL) activities according to the manufacturer’s instructions. The biochemical data was supplied within Supplementary File S1.

### 4.4. Histological Tissue Processing

The dissected brain tissues for routine histological examination were fixed in 10% formalin for 24 h. Following fixation, a series of processes including hydration with increasing alcohol concentrations (80%, 90%, 96% ethanol) and xylene (clearing) were applied. The tissues were embedded in paraffin blocks. Sections of 4–5 µm thickness were then obtained from the paraffin blocks for routine histological examination [31]. Sections were treated with a 3% hydrogen peroxide (H_2_O_2_) solution followed by a 20 min incubation. Subsequently, they were processed with Ultra V Block solution for 7 min. Primary antibodies for IBA-1 (sc-32725, Santa Cruz Biotechnology Inc., Dallas, TX, USA, diluted 1/100), NFκB (sc-8008, Santa Cruz Biotechnology Inc., Dallas, TX, USA, diluted 1/100), and IL-6 (sc-57315, Santa Cruz Biotechnology Inc., Dallas, TX, USA, diluted 1/100) were applied to the sections and then incubated overnight at +4 °C. Sections with biotinylated secondary antibodies were subjected to a 14 min treatment. Following this, sections treated with streptavidin-peroxidase were incubated for 15 min. Diaminobenzidine (DAB) was applied to the sections, and the resulting reaction was observed under a microscope. Subsequently, sections were counterstained with Harris hematoxylin. Samples were examined and imaged using a Zeiss Imager A2 photomicroscope (Microscope Axio Imager.A2, coded for transmitted-light bright field, tube 30°/23; Carl Zeiss Limited, Cambourne CB23 6DW, UK) [58].

### 4.5. Semi-Quantitative Analysis of IBA-1, NFκB, and IL6 Protein Expression Levels

The expression levels of IBA-1, NFκB, and IL6 proteins in brain tissue sections were evaluated using Image J software (version 1.53, http://imagej.nih.gov/, accessed on 20 May 2024). The measurements were conducted according to the protocol described by Crowe et al. [59]. Quantitative analysis was conducted by examining ten fields from each sample within each group. The presence of a brown color tone in the samples signified the positive expression of the targeted antibody, whereas the presence of a blue color tone indicated the negative expression of the antibody of interest. The signal intensity (expression) within a specific area was determined by dividing the expression of the target antibody by the total area of the specimen. For each section, signal intensity was calculated from the designated ten fields. Average values were calculated for the groups and analyzed for semi-quantitative immunohistochemistry scoring. Sections were imaged using the Zeiss Imager A2 light microscope [60].

### 4.6. Gene Ontology Analysis of Common Skimmianine and IBA-1, IL-6, and NF-κB Targets

To understand the potential biological processes in which skimmianine may be involved through IBA-1, IL-6, and NF-κB proteins, functional annotation analysis was conducted. Firstly, SwissTargetPrediction (http://www.swisstargetprediction.ch/, accessed on 10 May 2024), ChEMBL (https://www.ebi.ac.uk/chembl/, accessed on 10 May 2024), PubChem (https://pubchem.ncbi.nlm.nih.gov/, accessed on 10 May 2024), STITCH (http://stitch.embl.de/, accessed on 10 May 2024), and DrugBank (https://go.drugbank.com/, accessed on 10 May 2024) databases were screened to identify the proteins with which skimmianine might interact. The parameters were left as default, and *Homo sapiens* was selected as the organism. Subsequently, the 200 interactor proteins of IBA-1 (Uniprot ID: P55008), IL-6 (Uniprot ID: P05231), and NF-κB (Uniprot ID: Q04206) were obtained from the STRING database (https://string-db.org/, accessed on 10 May 2024). Then, the Venn diagram was constructed and the common interactors between skimmianine and the focused proteins were identified using the jvenn tool (https://jvenn.toulouse.inrae.fr/app/index.html, accessed on 10 May 2024) [61]. The shared proteins were subjected to Gene Ontology (GO) biological process analysis using the Enrichr platform (https://maayanlab.cloud/Enrichr/, accessed on 10 May 2024). Enrichments with a *p*-value less than 0.05 were considered significant and ranked based on their *p*-values. The semi-quantitative data was supplied within Supplementary File S2.

### 4.7. Statistical Analysis

Statistical analysis was performed using GraphPad Prism 7 (GraphPad Prism, San Diego, CA, USA) and SAS v9.3 (SAS Institute Inc., Cary, NC, USA) software programs. Shapiro–Wilk tests were performed to evaluate the normality of the data. Since the data were non-normally distributed, continuous variables were presented as median (quartile 1–quartile 3). Within-group comparisons were analyzed by the non-parametric Kruskal–Wallis test, followed by Dunn’s post hoc test. A significance level was considered as *p* < 0.05.

**Figure 6:**
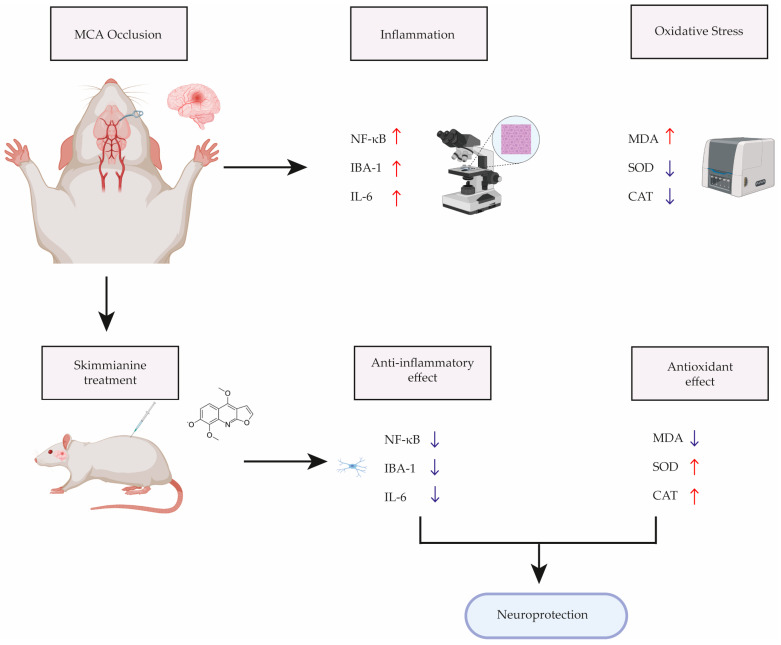
Illustrative summary of the experiment. Created with Biorender.com, accessed on 15 April 2024. Abbreviations: NF-κB: Nuclear factor kappa B, IBA-1: Ionized calcium-binding adapter molecule 1, IL-6: Interleukin-6, MDA: Malondialdehyde, SOD: Superoxide dismutase, CAT: Catalase.

Figure 6 shows major steps of experiments and summary of results obtained.

## 5. Conclusions

The findings of the study suggest that the antioxidant and anti-inflammatory effects of skimmianine on cerebral IR injury could be considered a potential therapeutic agent. However, further studies are needed to evaluate the clinical applicability of these findings. Future research could elucidate the specific molecular mechanisms underlying skimmianine’s modulation of inflammatory and antioxidant effects mediated by IBA-1, IL-6, and NF-κB. Exploring the potential synergistic interactions between skimmianine and other signaling pathways implicated in cerebral ischemia/reperfusion injury could offer valuable insights into enhancing its therapeutic efficacy.

## Data Availability

All generated data are presented in this study.

## Supplementary Materials

The following are available online at https://www.mdpi.com/article/10.3390/cimb46070437/s1, File S1: Skimmianine MDA, SOD and CAT biochemical data; File S2: Data of semi-quantitative Analysis of IBA-1, NF-κB, and IL6 Protein Expression.

## References

[1] Tirandi A., Sgura C., Carbone F., Montecucco F., Liberale L. (2023). Inflammatory biomarkers of ischemic stroke. Intern. Emerg. Med..

[2] Chen X., Yang T., Luo Y., Feng Z., Fang R., Ge J., Mei Z. (2023). Methodological and reporting quality evaluation of Buyang Huanwu decoction for experimental cerebral ischemia–reperfusion injury: A systematic review. Naunyn-Schmiedeberg’s Arch. Pharmacol..

[3] Fan Y., Luo Q., Wei J., Lin R., Lin L., Li Y., Chen Z., Lin W., Chen Q. (2017). Mechanism of salvianolic acid B neuroprotection against ischemia/reperfusion induced cerebral injury. Brain Res..

[4] L L., X W., Z Y. (2016). Ischemia-reperfusion Injury in the Brain: Mechanisms and Potential Therapeutic Strategies. Biochem. Pharmacol. Open Access.

[5] Kuriakose D., Xiao Z. (2020). Pathophysiology and Treatment of Stroke: Present Status and Future Perspectives. Int. J. Mol. Sci..

[6] Sun Y., Yang X., Xu L., Jia M., Zhang L., Li P., Yang P. (2023). The Role of Nrf2 in Relieving Cerebral Ischemia-Reperfusion Injury. Curr. Neuropharmacol..

[7] Balami J.S., Chen R.-L., Grunwald I.Q., Buchan A.M. (2011). Neurological complications of acute ischaemic stroke. Lancet Neurol..

[8] Campbell B.C.V., De Silva D.A., Macleod M.R., Coutts S.B., Schwamm L.H., Davis S.M., Donnan G.A. (2019). Ischaemic stroke. Nat. Rev. Dis. Prim..

[9] Magid-Bernstein J., Girard R., Polster S., Srinath A., Romanos S., Awad I.A., Sansing L.H. (2022). Cerebral Hemorrhage: Pathophysiology, Treatment, and Future Directions. Circ. Res..

[10] Campbell B.C.V., Khatri P. (2020). Stroke. Lancet.

[11] Zheng T., Jiang T., Huang Z., Ma H., Wang M. (2023). Role of traditional Chinese medicine monomers in cerebral ischemia/reperfusion injury:a review of the mechanism. Front. Pharmacol..

[12] Zhang M., Liu Q., Meng H., Duan H., Liu X., Wu J., Gao F., Wang S., Tan R., Yuan J. (2024). Ischemia-reperfusion injury: Molecular mechanisms and therapeutic targets. Signal Transduct. Target. Ther..

[13] Wu M.-Y., Yiang G.-T., Liao W.-T., Tsai A.P.Y., Cheng Y.-L., Cheng P.-W., Li C.-Y., Li C.J. (2018). Current Mechanistic Concepts in Ischemia and Reperfusion Injury. Cell. Physiol. Biochem..

[14] Pretzsch E., Nieß H., Ben Khaled N., Bösch F., Guba M., Werner J., Angele M., Chaudry I.H. (2022). Molecular Mechanisms of Ischaemia-Reperfusion Injury and Regeneration in the Liver-Shock and Surgery-Associated Changes. Int. J. Mol. Sci..

[15] Soares R.O.S., Losada D.M., Jordani M.C., Évora P., Castro-E-Silva O. (2019). Ischemia/Reperfusion Injury Revisited: An Overview of the Latest Pharmacological Strategies. Int. J. Mol. Sci..

[16] Imran R., A Mohamed G., Nahab F. (2021). Acute Reperfusion Therapies for Acute Ischemic Stroke. J. Clin. Med..

[17] Bhaskar S., Stanwell P., Cordato D., Attia J., Levi C. (2018). Reperfusion therapy in acute ischemic stroke: Dawn of a new era?. BMC Neurol..

[18] Wang Y., Xiao G., He S., Liu X., Zhu L., Yang X., Zhang Y., Orgah J., Feng Y., Wang X. (2020). Protection against acute cerebral ischemia/reperfusion injury by QiShenYiQi via neuroinflammatory network mobilization. Biomed. Pharmacother..

[19] Huang D., Cao Y., Zu T., Ju J. (2021). Interference with long noncoding RNA SNHG3 alleviates cerebral ischemia-reperfusion injury by inhibiting microglial activation. J. Leukoc. Biol..

[20] Przykaza Ł. (2021). Understanding the Connection Between Common Stroke Comorbidities, Their Associated Inflammation, and the Course of the Cerebral Ischemia/Reperfusion Cascade. Front. Immunol..

[21] Atanasov A.G., Zotchev S.B., Dirsch V.M., Supuran C.T. (2021). Natural products in drug discovery: Advances and opportunities. Nat. Rev. Drug Discov..

[22] Yu L., Zhang Y., Chen Q., He Y., Zhou H., Wan H., Yang J. (2022). Formononetin protects against inflammation associated with cerebral ischemia-reperfusion injury in rats by targeting the JAK2/STAT3 signaling pathway. Biomed. Pharmacother..

[23] Shen H., Pei H., Zhai L., Guan Q., Wang G. (2022). Salvianolic acid C improves cerebral ischemia reperfusion injury through suppressing microglial cell M1 polarization and promoting cerebral angiogenesis. Int. Immunopharmacol..

[24] Yang X., Xu L., Zhao H., Xie T., Wang J., Wang L., Yang J. (2023). Curcumin protects against cerebral ischemia-reperfusion injury in rats by attenuating oxidative stress and inflammation: A meta-analysis and mechanism exploration. Nutr. Res..

[25] Liu Y., Zhu X., Tong X., Tan Z. (2021). Syringin protects against cerebral ischemia/reperfusion injury via inhibiting neuroinflammation and TLR4 signaling. Perfusion.

[26] Kawadkar M., Mandloi A.S., Saxena V., Tamadaddi C., Sahi C., Dhote V.V. (2021). Noscapine alleviates cerebral damage in ischemia-reperfusion injury in rats. Naunyn-Schmiedeberg’s Arch. Pharmacol..

[27] Liu Y., Kang L., Shi S.-M., Li B.-J., Zhang Y., Zhang X.-Z., Guo X.-W., Fu G., Zheng G.-N., Hao H. (2022). Skimmianine as a novel therapeutic agent suppresses proliferation and migration of human esophageal squamous cell carcinoma via blocking the activation of ERK1/2. Neoplasma.

[28] Huo C.-L., Wang B., Zhang X., Sun Z.-G. (2023). Skimmianine attenuates liver ischemia/reperfusion injury by regulating PI3K–AKT signaling pathway-mediated inflammation, apoptosis and oxidative stress. Sci. Rep..

[29] Sabu V., Krishnan S., Peter J., Aswathy I., Preethi S.L., Simon M., Radhakrishna G.P., Helen A. (2021). Synergistic effect of Betulinic acid, Apigenin and Skimmianine (BASk) in high cholesterol diet rabbit: Involvement of CD36-TLR2 signaling pathway. Cytokine.

[30] Ogunrinade F.A., Iwuanyanwu V.U., Sarker S.D., Olajide O.A. (2023). Neuroprotection by Skimmianine in Lipopolysaccharide-Activated BV-2 Microglia. Molecules.

[31] Hopperton K.E., Mohammad D., Trépanier M.O., Giuliano V., Bazinet R.P. (2018). Markers of microglia in post-mortem brain samples from patients with Alzheimer’s disease: A systematic review. Mol. Psychiatry.

[32] He Y., Ma X., Li D., Hao J. (2016). Thiamet G mediates neuroprotection in experimental stroke by modulating microglia/macrophage polarization and inhibiting NF-κB p65 signaling. J. Cereb. Blood Flow Metab..

[33] Shih R.-H., Wang C.-Y., Yang C.-M. (2015). NF-kappaB Signaling Pathways in Neurological Inflammation: A Mini Review. Front. Mol. Neurosci..

[34] Chen Y., Zhang C., Zhao L., Chen R., Zhang P., Li J., Zhang X., Zhang X. (2024). Eriocalyxin B alleviated ischemic cerebral injury by limiting microglia-mediated excessive neuroinflammation in mice. Exp. Anim..

[35] Zhu H., Hu S., Li Y., Sun Y., Xiong X., Hu X., Chen J., Qiu S. (2022). Interleukins and Ischemic Stroke. Front. Immunol..

[36] Chen Y., Wu J., Zhu J., Yang G., Tian J., Zhao Y., Wang Y. (2021). Artesunate Provides Neuroprotection against Cerebral Ischemia–Reperfusion Injury via the TLR-4/NF-κB Pathway in Rats. Biol. Pharm. Bull..

[37] Shi C., Li J., Li J. (2020). Ephedrine attenuates cerebral ischemia/reperfusion injury in rats through NF-κB signaling pathway. Hum. Exp. Toxicol..

[38] Zhang C., Ma Y., Zhao Y., Guo N., Han C., Wu Q., Mu C., Zhang Y., Tan S., Zhang J. (2024). Systematic review of melatonin in cerebral ischemia-reperfusion injury: Critical role and therapeutic opportunities. Front. Pharmacol..

[39] Li Z.-W., Tang H., Chen X.-X., Li X.-X., Xu H.-H., Chen M.-H., Ba H.-J., Lin Q., Dai J.-X., Cai J.-Y. (2024). Urolithin B Attenuates Cerebral Ischemia–reperfusion Injury by Modulating Nrf2-regulated Anti-oxidation in Rats. Neuroscience.

[40] Ratheesh M., Sindhu G., Helen A. (2013). Anti-inflammatory effect of quinoline alkaloid skimmianine isolated from *Ruta graveolens* L. Inflamm. Res..

[41] Hao T., Yang Y., Li N., Mi Y., Zhang G., Song J., Liang Y., Xiao J., Zhou D., He D. (2020). Inflammatory mechanism of cerebral ischemia-reperfusion injury with treatment of stepharine in rats. Phytomedicine.

[42] Liu R., Xu N.-G., Yi W., Ji C. (2020). Electroacupuncture Attenuates Inflammation after Ischemic Stroke by Inhibiting NF-*κ*B-Mediated Activation of Microglia. Evidence-Based Complement. Altern. Med..

[43] Yang S., Wang H., Yang Y., Wang R., Wang Y., Wu C., Du G. (2019). Baicalein administered in the subacute phase ameliorates ischemia-reperfusion-induced brain injury by reducing neuroinflammation and neuronal damage. Biomed. Pharmacother..

[44] Zhao B., Shi Q., Zhang Z., Wang S., Wang X., Wang H. (2018). Protective effects of paeonol on subacute/chronic brain injury during cerebral ischemia in rats. Exp. Ther. Med..

[45] Chu K., Yin B., Wang J., Peng G., Liang H., Xu Z., Du Y., Fang M., Xia Q., Luo B. (2012). Inhibition of P2X7 receptor ameliorates transient global cerebral ischemia/reperfusion injury via modulating inflammatory responses in the rat hippocampus. J. Neuroinflamm..

[46] Xiang B., Zhong P., Fang L., Wu X., Song Y., Yuan H. (2019). miR-183 inhibits microglia activation and expression of inflammatory factors in rats with cerebral ischemia reperfusion via NF-κB signaling pathway. Exp. Ther. Med..

[47] Yang Z.F., Ho D.W., Lau C.K., Lam C.T., Lum C.T., Poon R.T.P., Fan S.T. (2005). Allograft inflammatory factor-1 (AIF-1) is crucial for the survival and pro-inflammatory activity of macrophages. Int. Immunol..

[48] Kadoya M., Yamamoto A., Hamaguchi M., Obayashi H., Mizushima K., Ohta M., Seno T., Oda R., Fujiwara H., Kohno M. (2014). Allograft inflammatory factor-1 stimulates chemokine production and induces chemotaxis in human peripheral blood mononuclear cells. Biochem. Biophys. Res. Commun..

[49] Elizondo D.M., Andargie T.E., Yang D., Kacsinta A.D., Lipscomb M.W. (2017). Inhibition of Allograft Inflammatory Factor-1 in Dendritic Cells Restrains CD4+ T Cell Effector Responses and Induces CD25+Foxp3+ T Regulatory Subsets. Front. Immunol..

[50] De Leon-Oliva D., Garcia-Montero C., Fraile-Martinez O., Boaru D.L., García-Puente L., Rios-Parra A., Garrido-Gil M.J., Casanova-Martín C., García-Honduvilla N., Bujan J. (2023). AIF1: Function and Connection with Inflammatory Diseases. Biology.

[51] Aliyu M., Zohora F.T., Anka A.U., Ali K., Maleknia S., Saffarioun M., Azizi G. (2022). Interleukin-6 cytokine: An overview of the immune regulation, immune dysregulation, and therapeutic approach. Int. Immunopharmacol..

[52] Borrás C., Gambini J., López-Grueso R., Pallardó F.V., Viña J. (2010). Direct antioxidant and protective effect of estradiol on isolated mitochondria. Biochim. et Biophys. Acta (BBA) Mol. Basis Dis..

[53] Clusan L., Ferrière F., Flouriot G., Pakdel F. (2023). A Basic Review on Estrogen Receptor Signaling Pathways in Breast Cancer. Int. J. Mol. Sci..

[54] Nilsson S., Mäkelä S., Treuter E., Tujague M., Thomsen J., Andersson G., Enmark E., Pettersson K., Warner M., Gustafsson J.A. (2001). Mechanisms of Estrogen Action. Physiol. Rev..

[55] Gruber C.J., Tschugguel W., Schneeberger C., Huber J.C. (2002). Production and Actions of Estrogens. New Engl. J. Med..

[56] A Walf A., A Frye C. (2006). A Review and Update of Mechanisms of Estrogen in the Hippocampus and Amygdala for Anxiety and Depression Behavior. Neuropsychopharmacology.

[57] Upadhayay S., Gupta R., Singh S., Mundkar M., Singh G., Kumar P. (2022). Involvement of the G-Protein-Coupled Estrogen Receptor-1 (GPER) Signaling Pathway in Neurodegenerative Disorders: A Review. Cell. Mol. Neurobiol..

[58] Keşim D.A., Aşır F., Ayaz H., Korak T. (2024). The Effects of Ellagic Acid on Experimental Corrosive Esophageal Burn Injury. Curr. Issues Mol. Biol..

[59] Crowe A.R., Yue W. (2019). Semi-quantitative Determination of Protein Expression Using Immunohistochemistry Staining and Analysis: An Integrated Protocol. Bio-Protocol.

[60] Aşır F., Oğlak S.C., Ağaçayak E., Alabalık U. (2023). Homeobox A Cluster 7 (HOXA7) protein expression increased in the placentas of patients with preterm delivery. Périnat. J..

[61] Bardou P., Mariette J., Escudié F., Djemiel C., Klopp C. (2014). jvenn: An interactive Venn diagram viewer. BMC Bioinform..

